# Global Public Health Surveillance under New International Health Regulations

**DOI:** 10.3201/eid1207.051497

**Published:** 2006-07

**Authors:** Michael G. Baker, David P. Fidler

**Affiliations:** * Wellington School of Medicine and Health Sciences, Wellington, New Zealand;; † Indiana University School of Law, Bloomington, Indiana, USA

**Keywords:** Disease surveillance, International law, Infectious disease, Emergence, Health law, International Health Regulations, Outbreaks, World Health Organization

## Abstract

The new International Health Regulations adopted by the World Health Assembly in May 2005 (IHR 2005) represents a major development in the use of international law for public health purposes. One of the most important aspects of IHR 2005 is the establishment of a global surveillance system for public health emergencies of international concern. This article assesses the surveillance system in IHR 2005 by applying well-established frameworks for evaluating public health surveillance. The assessment shows that IHR 2005 constitutes a major advance in global surveillance from what has prevailed in the past. Effectively implementing the IHR 2005 surveillance objectives requires surmounting technical, resource, governance, legal, and political obstacles. Although IHR 2005 contains some provisions that directly address these obstacles, active support by the World Health Organization and its member states is required to strengthen national and global surveillance capabilities.

## Abstract 2

IHR 2005 establishes a global surveillance system for public health emergencies of international concern.

On May 23, 2005, the World Health Assembly adopted the new International Health Regulations (IHR 2005) (*1*) as an international treaty. This step concluded the decade-long effort led by the World Health Organization (WHO) to revise the old regulations (IHR 1969) to make them more effective against global disease threats. Originally adopted in 1951 (*2*) and last substantially changed in 1969 (*3*), IHR 1969 had lost its effectiveness and relevance by the mid-1990s, if not earlier (*4*).

The resurgence of infectious diseases noted in the first half of the 1990s showed IHR 1969's limitations. For example, after smallpox was eradicated in the late 1970s, IHR 1969 only applied to the traditionally "quarantinable" diseases of cholera, plague, and yellow fever. In addition, IHR 1969 restricted surveillance to information provided only by governments, lacked mechanisms for swiftly assessing and investigating public health risks, contained no strategies for developing surveillance capacities and infrastructure, and failed to generate compliance by WHO member states. WHO began revising IHR 1969 in 1995 (*5*), and IHR 2005's adoption completed the modernization of this important body of international law on public health.

IHR 2005 departs radically from IHR 1969 and represents a historic development in international law on public health (*6*). IHR 2005 expands the scope of the regulations' application, strengthens WHO's authority in surveillance and response, contains more demanding surveillance and response obligations, and applies human rights principles to public health interventions. The most dramatic of these changes involves a new surveillance system that far surpasses what the IHR 1969 contained. After reviewing key surveillance concepts and frameworks, this article describes IHR 2005's surveillance regime and assesses its likely performance. It concludes by discussing obstacles that could prevent IHR 2005 from becoming an effective global public health surveillance system and addressing how these obstacles might be overcome.

## Key Surveillance Concepts and Evaluation Framework

Public health surveillance has been defined as "the ongoing systematic collection, analysis, and interpretation of outcome-specific data for use in the planning, implementation, and evaluation of public health practice" (*7*). A surveillance system requires structures and processes to support these ongoing functions (*7*).

The Centers for Disease Control and Prevention (CDC) developed guidelines that identify the essential elements and attributes for an effective public health surveillance system (*8*). According to these guidelines, evaluating surveillance systems involves 2 main steps: 1) describing the purpose, operation, and elements of the system and 2) assessing its performance according to key attributes. This article uses this 2-step approach to evaluate the global public health surveillance system prescribed by IHR 2005.

## Surveillance System Specified in IHR 2005

In the CDC framework, describing a surveillance system includes 4 main elements: 1) health-related events under surveillance and their public health importance, 2) purpose and objectives of the system, 3) components and processes of the system, and 4) resources needed to operate it (*8*).

### Health-related Events under Surveillance

IHR 2005 identifies health-related events that each country that agrees to be bound by the regulations (a "state party") must report to WHO. In terms of health-related events that occur in its territory, a state party must notify WHO of "all events which may constitute a public health emergency of international concern" (article 6.1). These events include any unexpected or unusual public health event regardless of its origin or source (article 7). IHR 2005 also requires state parties, as far as is practicable, to inform WHO of public health risks identified outside their territories that may cause international disease spread, as manifested by exported or imported human cases, vectors that may carry infection or contamination, or contaminated goods (article 9.2).

IHR 2005 provides guidance to assist state parties' compliance with these obligations in 4 ways. First, IHR 2005 defines a "public health emergency of international concern" (PHEIC) as "an extraordinary event which is determined [by the WHO Director-General]… (i) to constitute a public health risk to other States through the international spread of disease and (ii) to potentially require a coordinated international response" (article 1.1). Unlike IHR 1969's limited scope of application to just 3 communicable diseases (*3*), IHR 2005 defines disease as an illness or medical condition that does or could threaten human health regardless of its source or origin (article 1.1). This scope therefore encompasses communicable and noncommunicable disease events, whether naturally occurring, accidentally caused, or intentionally created.

**Figure 1:**
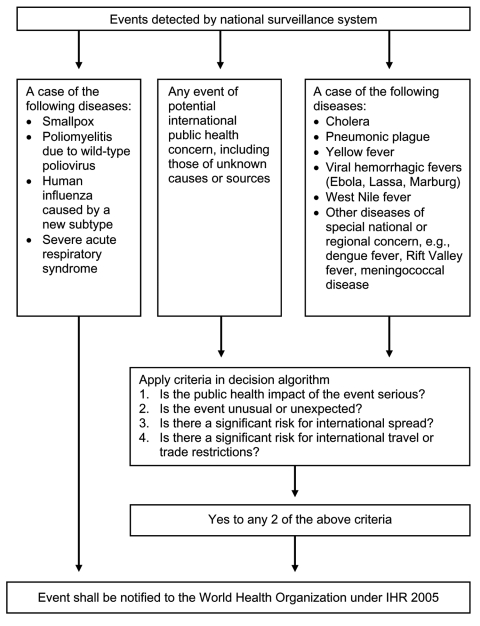
International Health Regulations (IHR) 2005 decision instrument (simplified from annex 2 of IHR).

Second, IHR 2005 contains a "decision instrument" (annex 2) that helps state parties identify whether a health-related event may constitute a PHEIC and therefore requires formal notification to WHO (Figure 1). The decision instrument focuses on risk assessment criteria of public health importance, including the seriousness of the public health impact and the likelihood of international spread.

Third, IHR 2005 includes a list of diseases for which a single case may constitute a PHEIC and must be reported to WHO immediately. This list consists of smallpox, poliomyelitis, human influenza caused by new subtypes, and severe acute respiratory syndrome (SARS). A second list of diseases exists (Figure 1) for which a single case requires the decision instrument to be used to assess the event, but notification is determined by the assessment and is not automatic. Finally, IHR 2005 also encourages state parties to consult with WHO over events that do not meet the criteria for formal notification but may still be of public health relevance (article 8).

IHR 2005's expansion of the range of public health events under surveillance and the use of risk assessment criteria in deciding what is reportable is possibly the single most important surveillance advance in IHR 2005. This change greatly enhances effective surveillance of emerging infectious diseases, which are "infections that have newly appeared in a population or have existed but are rapidly increasing in incidence or geographic range" (*9*). IHR 2005's surveillance strategy, especially the decision instrument, has been specifically designed to make IHR 2005 directly applicable to emerging infectious disease events, which are usually unexpected and often threaten to spread internationally.

In addition to events that may constitute a PHEIC, IHR 2005 also requires state parties to report the health measures (e.g., border screening, quarantine) that they implement in response to such events (article 6). State parties are also specifically required to inform WHO within 48 hours of implementing additional health measures that interfere with international trade and travel, unless the WHO Director-General has recommended such measures (article 43).

### Purpose and Objectives of Surveillance under IHR 2005

IHR 2005's purpose is to prevent, protect against, control, and facilitate public health responses to the international spread of disease (article 2), and IHR 2005 makes surveillance central to guiding effective public health action against cross-border disease threats. The regulations define surveillance as "the systematic ongoing collection, collation and analysis of data for public health purposes and the timely dissemination of public health information for assessment and public health response as necessary" (article 1.1). Surveillance is central to IHR 2005's public health objectives, which explains why IHR 2005 requires all state parties to develop, strengthen, and maintain core surveillance capacities (article 5.1). This obligation goes beyond anything concerning surveillance in IHR 1969, which did not address surveillance infrastructure and capabilities beyond a general requirement for a state party to notify WHO of any outbreak of a disease subject to the regulations.

### Components and Processes of IHR 2005 Surveillance

**Figure 2:**
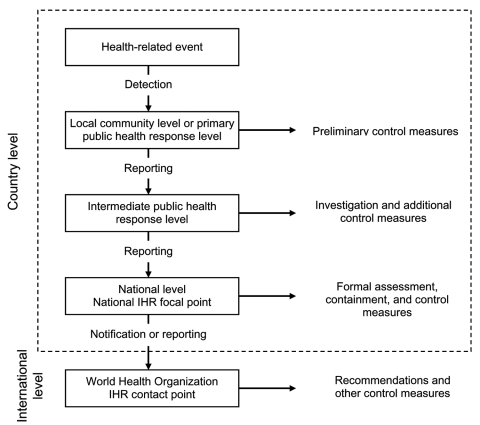
Public health surveillance structures and processes specified in International Health Regulations (IHR) 2005.

IHR 2005 describes key aspects of the surveillance process from the local to the global level. As part of IHR 2005's core surveillance and response capacity requirements, each state party has to develop and maintain capabilities to detect, assess, and report disease events at the local, intermediate, and national levels (article 5.1, annex 1). Officials at the national level must be able to report through the national IHR focal point to WHO when required under IHR 2005 (articles 4.2 and 6). The regulations also mandate that WHO establish IHR contact points that are always accessible to state parties (article 4.3). Connecting these levels produces the surveillance architecture illustrated in Figure 2.

Requiring that a national IHR focal point be established is another surveillance initiative in IHR 2005. The focal point is designed to facilitate rapid sharing of surveillance information because it is responsible for communicating with the WHO IHR contact points and disseminating information within the state party (article 4.2). By linking national IHR focal points through WHO, IHR 2005 establishes a global network that improves the real-time flow of surveillance information from the local to the global level and also between state parties (article 4.4).

### Resources Needed to Operate IHR 2005's Surveillance System

Building and maintaining the surveillance system envisioned in IHR 2005 will require substantial financial and technical resources. State parties will be primarily responsible for providing resources needed to develop their core surveillance capacities. Each state party has to assess its ability to meet the core surveillance requirements by June 2009. In addition, each state party has to develop and implement a plan for ensuring compliance with core surveillance obligations (articles 5.1 and 5.2, annex 1).

WHO is obliged to assist state parties in meeting their surveillance system obligations (article 5.3), but this provision does not allocate any WHO funds for this purpose. State parties are required to collaborate with each other in providing technical cooperation and logistical support for surveillance capabilities and in mobilizing financial resources to facilitate implementation of IHR 2005 (article 44.1).

## Evaluating the IHR 2005 Surveillance System's Attributes and Potential Performance

Key attributes of effective surveillance systems identified by CDC are usefulness, sensitivity, timeliness, stability, simplicity, flexibility, acceptability, data quality, positive predictive value, and representativeness. Of these attributes, usefulness, sensitivity, timeliness, and stability will be most critical to the success of the IHR 2005 surveillance system. Simplicity, acceptability, and flexibility will affect the establishment and sustainability of the surveillance system. Data quality, positive predictive value, and representativeness are central to accurately characterizing health-related events under surveillance. Table 1 summarizes these attributes, provides commentary on their relevance to effective surveillance under IHR 2005, and assesses the likely performance of the IHR 2005 surveillance system for each attribute. The following paragraphs concentrate on assessing IHR 2005 with respect to the key attributes of usefulness, sensitivity, timeliness, and stability.

**Table 1:** International Health Regulations (IHR 2005) assessed according to attributes of public health surveillance systems (adapted from [8])

Attribute	Attribute details	Relevance to IHR 2005
Usefulness	Contribution to prevention and control of adverse health-related events	Design and scope imply improved usefulness compared with IHR 1969 but attribute must be evaluated after IHR 2005 has operated for a period
Sensitivity	Proportion of true events detected by system and ability to detect outbreaks	Specifies notification of all potential public health emergencies of international concern (PHEIC) and provides multiple pathways to increase sensitivity
Timeliness	Speed between steps particularly from event onset to response	Specifies assessment within 48 h and reporting within 24 h by state parties and prescribes immediate reporting of events at local and intermediate levels within state parties
Stability	Reliability and availability of surveillance system	All state parties must notify all potential PHEIC from June 2007 and establish capacity to detect, assess, and report events by 2012, with potential extensions to 2016
Simplicity	Simplicity of structure and ease of operation	Architecture of surveillance system is streamlined and transparent, especially at international level
Flexibility	Ability to adapt to changing information needs and operating conditions	Use of risk assessment criteria means that surveillance applies to new as well as established disease threats
Acceptability	Willingness of persons and organizations to participate	Establishment of surveillance in international law represents commitment by state parties to participate
Data quality	Completeness and validity of recorded data	Specifies information to be reported and includes provisions for validation and assessment of all reports to separate rumors from real events
Positive predictive value	Proportion of reported events that are true events	Oriented toward high sensitivity with correspondingly low specificity and positive predictive value, so WHO will not declare most notified events to be PHEIC
Representativeness	Ability to describe events over time and their distribution by place and person	Likely to be increased after validation and assessment, as for data quality

### Usefulness of the Surveillance System

The central premise of IHR 2005 is that rapidly detecting PHEIC will support improved disease prevention and control both within and between state parties. Ample evidence shows that delayed recognition and response to emerging diseases may result in adverse consequences in terms of illness and death, spread to other countries, and disruption of trade and travel (*10*). The usefulness of surveillance under IHR 2005 represents the sum of all the critical system attributes and can only be assessed after the system is in operation, so this attribute is not discussed here. However, for the future sustainability and development of IHR 2005, we must evaluate its overall usefulness and document its contribution to prevention and control of adverse health events. IHR includes mechanisms to review and, if necessary, amend its provisions and in particular requires periodic evaluation of the functioning of the decision instrument (article 54).

### Sensitivity of the Surveillance System

The IHR 2005 surveillance provisions imply 100% sensitivity as a standard, namely the reporting of all events that meet notification requirements. The use of risk assessment criteria (Figure 1) also allows for higher sensitivity for PHEIC than would be possible with a list of predetermined disease threats (as in IHR 1969). To test the potential sensitivity of the decision instrument proposed in drafts of the revised IHR in 2004, investigators in the United Kingdom applied the then-proposed decision instrument to all events (N = 30) that were important enough to have been published in the national surveillance bulletin for England and Wales during 2003 (*11*). According to this method, 12 of the 30 events would have been reportable under the decision instrument. These events included all those that were considered potential PHEIC. Investigators concluded that the decision instrument was highly sensitive for selecting outbreaks and incidents that require reporting under the proposed IHR revision.

The sensitivity of the IHR 2005 surveillance system will probably be affected by 2 factors. First, in all likelihood, inadequate capacities at the local and intermediate levels within state parties will limit the system's sensitivity more than capacities at the national level. Second, state parties may not always be willing to comply with their reporting obligations in the face of possible adverse political and economic consequences that may result from alerting the world to a disease event in their territories. Fear of such adverse consequences undermined reporting obligations in IHR 1969.

IHR 2005 incorporates strategies to address these potential limitations. First, as noted above, IHR 2005 requires state parties to build and maintain core local, intermediate, and national surveillance capabilities (article 5.1, annex 1). Fulfillment of this obligation will improve surveillance capacity vertically, from local to national levels, which should support higher sensitivity.

Second, IHR 2005 permits WHO to improve sensitivity by collecting and using information from multiple sources. IHR 1969 only allowed WHO to use information provided by state parties (*3*), and failure of state parties to abide by their reporting obligations adversely affected WHO surveillance activities (*5*). Under IHR 2005, WHO can collect, analyze, and use information gathered from governments, other intergovernmental organizations, and nongovernmental organizations and actors (article 9.1). By permitting WHO to cast its surveillance network beyond information it receives from governments, IHR 2005 creates opportunities for WHO to improve the sensitivity of the surveillance system and avoid being blocked by governmental failure to comply with reporting requirements.

### Timeliness of the Surveillance System

Public health practitioners understand how timely notification of public health risks is necessary for effective intervention strategies (*12**,**13*), lessons reiterated in the SARS pandemic (*14*). Timely surveillance is also stressed in connection with strategies to deal with pandemic influenza (*15**,**16*). Timeliness may be the most important attribute that IHR 2005 will have to demonstrate to be effective.

IHR 2005 contains several provisions that relate to timeliness. National-level assessments with the decision instrument must be completed within 48 hours (annex 1, part A, 6[a]). State parties must then notify WHO within 24 hours of assessing any event that may constitute a PHEIC or that is unexpected or unusual (articles 6.1 and 7). The same 24-hour requirement applies to reporting public health risk outside a state party's territory that may constitute a PHEIC (article 9). State parties must also respond within 24 hours to all requests that WHO makes for verification of health-related events in their territories (article 10.2).

Timeliness of reporting is likely to be affected more by actions taken at local and intermediate levels than national-level provision of information to WHO. In this regard, IHR 2005 includes the core surveillance capacity that local and intermediate public health entities must be able to carry out their reporting responsibilities immediately (annex 1).

WHO's ability to draw on a wide array of sources of information, including the Internet and nongovernmental organizations and actors, may enhance the timeliness of the IHR 2005 surveillance system (*13**,**17*). In countries that have less well-developed local, intermediate, and national surveillance systems, nongovernmental sources of information can often provide information faster than governments. Accessing this type of information early and often helps WHO contact countries sooner, which increases the chances of more effective interventions.

### Stability of the Surveillance System

The obligations each state party has to build and maintain core capacities in surveillance at the local, intermediary, and national levels, combined with the responsibilities for surveillance WHO has globally, should construct a global surveillance system that will be stable and reliable over time. Recognizing that core capacities at the national level and below will not develop overnight, IHR 2005 gives state parties until June 2012 to develop these capacities (article 5.1). State parties can obtain a 2-year extension on this deadline by submitting a justified need and an implementation plan and can request an additional 2-year extension, which the WHO Director-General has the discretion to approve or deny (article 5.2).

The 5-year grace period, and the possibility of 2-year extensions, was a necessary compromise and reflects the difficulties many developing states will have in improving their surveillance systems. The stability and reliability of the IHR 2005 surveillance system are designed to increase steadily as the grace period and any extensions come to an end.

## Potential Obstacles to Achieving IHR 2005 Surveillance System Objectives

Continued lamentations about the weaknesses of public health surveillance nationally and globally (*18*) illustrate that achieving useful, sensitive, timely, and stable surveillance through IHR 2005 will be a challenge for states and the international community. Several potential obstacles, including technical, resource, governance, legal, and political concerns, will complicate and frustrate efforts to improve national and global surveillance capabilities. Table 2 summarizes these potential barriers and possible responses.

**Table 2:** Barriers to International Health Regulations (IHR) 2005 surveillance effectiveness, and potential responses

Barrier	Description	Potential responses
Technical	Difficulty detecting previously unrecognized pathogens, especially those with asymptomatic transmission	Specialized surveillance approaches such as syndromic surveillance; improved diagnostic technologies; training and support for epidemiology, laboratory, and other staff
Resource	Limited resources for public health surveillance, particularly in developing countries	Systematic global strategy for assessment and development of surveillance and response capacities, particularly in developing countries
Governance	Lack of awareness about limitations of existing surveillance and lack of governance capabilities to develop and manage sophisticated systems	Training and support for public health professionals and managers; periodic surveillance system evaluations; performance monitoring focusing on attributes such as sensitivity and timeliness
Legal	Potential for countries to make reservations to some obligations in IHR 2005 and concerns it may not be consistent with domestic law in some countries	Formulation of reservations to ensure minimal effects on public health surveillance; development of "model" public health legislation that can be adapted for use in many countries
Political	Concern about potential negative effects on trade and tourism from reporting disease events	Strategies to limit excessive responses; fostering a collaborative, measured response to public health emergencies of international concern; awareness of self-defeating effects of withholding information

### Technical Issues

Emerging infectious diseases often create technical challenges for surveillance, even for the most technologically advanced and well-resourced countries. The sensitivity of surveillance systems for new pathogens has historically been limited, particularly if such pathogens presented themselves in unusual or unexpected ways. Recent modeling has shown that the ability to control the spread of a new pathogen is influenced by the proportion of transmission that occurs before the onset of overt symptoms or through asymptomatic infection (*19*). This property explains why diseases such as influenza and HIV may be more difficult to control than smallpox or SARS.

Consequently, surveillance needs to be sufficiently sensitive to detect infectious agents that have not yet resulted in large numbers of diagnosed cases. One approach to this challenge is syndromic surveillance (*20*), but such surveillance has not been effective in detecting emerging infectious diseases early (*21*). In fact, WHO abandoned syndromic surveillance as a strategy for the revised IHR after pilot studies demonstrated that it was not effective (*22*). Improved diagnostic technologies may also help public health authorities identify new pathogenic threats (*23*). Strategies for enhancing reporting processes have been well described (*24*).

### Resource Issues

The demands of IHR 2005 surveillance obligations will confront many countries, particularly developing countries, with resource challenges. IHR 2005 does not include financing mechanisms, which leaves each state party to bear the financial costs of improving its own local, intermediate, and national level surveillance capabilities. The obligation on state parties and WHO to collaborate in mobilizing financial resources (article 44) is a weak obligation at best. The lack of economic resources will, if not more vigorously addressed as recommended by the UN Secretary-General (*25*), retard progress on all aspects of the upgraded surveillance system. WHO, in conjunction with the United Nations and the World Bank, could consider developing a global strategy to support the development and maintenance of core surveillance capacities.

### Governance Issues

Governance obstacles include managerial and administrative weaknesses in countries from the local to the national level. Few countries have conducted a systematic review of their surveillance systems, and thus most lack detailed knowledge of gaps and limitations in their surveillance infrastructures and how to address these problems (*26*). Only a few states have assessed their ability to detect and respond to emerging disease threats, such as those posed by bioterrorism agents (*27*). The IHR 2005 requirement that each state party assess the condition of its public health surveillance within 2 years of the regulations' entry into force should help countries improve their national governance for surveillance purposes. Again, many states will need external assistance with such work.

### Legal Issues

State parties may face legal complications in implementing IHR 2005 within their national legal and constitutional systems. For example, the United States has indicated that requirements of US federalism may affect its compliance with IHR 2005 (*28*). The US position suggests that other countries may also wish to formulate reservations to IHR 2005 to account for the demands of their national constitutional structures and systems of law (*29*). Whether such reservations will undermine the IHR 2005 surveillance system cannot be assessed, but this concern has to be monitored closely as countries determine whether reservations are required under their national constitutional systems. IHR 2005 also specifies that domestic legislation and administrative arrangements be adjusted fully with IHR 2005 by June 2007, or by June 2008 after a suitable declaration to the WHO Director-General (article 59.3). Helping state parties update their public health law may be technical assistance that industrialized countries can provide.

### Political Issues

Questions remain about the level of political commitment countries will demonstrate in implementing IHR 2005. IHR 1969 suffered because state parties frequently failed to report notifiable diseases and routinely applied excessive trade and travel restrictions (*4*). The relevance of such trade and travel concerns was most recently illustrated during the SARS pandemic through China's initial fears that disclosing the pandemic would harm its economy and foreign trade (*30**,**31*). WHO's access to nongovernmental sources of surveillance information reduces the incentives that state parties once had to hide disease events, as was demonstrated during the SARS pandemic (*32*). In addition, IHR 2005 includes provisions that require WHO to recommend, and state parties to use, control measures that are no more restrictive than necessary to achieve the desired level of health protection (articles 17, 43). Uncertainty lingers, however, as to whether these obligations will fare better in terms of state party compliance than similar ones in IHR 1969.

## Conclusion

Establishing effective global public health surveillance is at the heart of IHR 2005. Evaluating the surveillance system specified by IHR 2005 is necessary to understand the potential for this new set of international legal rules to contribute to global health governance. IHR 2005 prescribes essential elements of a surveillance system and seeks to achieve the critical attributes of usefulness, sensitivity, timeliness, and stability. These features resonate with other aspects of IHR 2005 that make it a seminal development for global health governance. In May 2006, the World Health Assembly adopted a resolution urging WHO member states to comply immediately, on a voluntary basis, with IHR 2005 in light of the threat posed by avian influenza (*33*).

The task of turning the IHR 2005 vision of an effective global public health surveillance system into reality is daunting. Of the obstacles complicating this challenge, lack of financial resources to upgrade surveillance systems, especially in developing countries, will be the most difficult to overcome. In IHR 2005, public health has been given a governance regime unlike anything in the history of international law on public health. Turning the blueprint detailed in IHR 2005 into functional architecture that benefits all is one of the great public health challenges of the first decades of the 21st century.
